# Ghrelin: Central Nervous System Sites of Action in Regulation of Energy Balance

**DOI:** 10.1155/2010/616757

**Published:** 2010-02-15

**Authors:** Mark Fry, Alastair V. Ferguson

**Affiliations:** ^1^Department of Biological Sciences, University of Manitoba, Winnipeg, MB, Canada R3T 2N2; ^2^Department of Physiology, Queen's University, Kingston, ON, Canada K7L 3N6

## Abstract

Ghrelin, a peptide hormone secreted by the stomach, has been shown to regulate energy homeostasis by modulating electrical activity of neurons in the central nervous system (CNS). Like many circulating satiety signals, ghrelin is a peptide hormone and is unable to cross the blood-brain barrier without a transport mechanism. In this review, we address the notion that the arcuate nucleus of the hypothalamus is the only site in the CNS that detects circulating ghrelin to trigger orexigenic responses. We consider the roles of a specialized group of CNS structures called the sensory circumventricular organs (CVOs), which are not protected by the blood-brain barrier. These areas include the subfornical organ and the area postrema and are already well known to be key areas for detection of other circulating hormones such as angiotensin II, cholecystokinin, and amylin. A growing body of evidence indicates a key role for the sensory CVOs in the regulation of energy homeostasis.

## 1. Introduction

Prior to 1999, the growth hormone secretagogue receptor (GHS-R) was an orphan receptor, known to evoke the release of growth hormone via a pathway independent of GHRH. The work of Kojima et al. [1] identified ghrelin, a 28-amino acid peptide secreted by the stomach, as the endogenous ligand for the GHS-R. Several facts immediately suggested that ghrelin might be involved in regulation of energy balance. First, ghrelin was secreted by the X/A-like cells in the fundus of the stomach and secretion was elevated with fasting [2], thus providing a clear signal from the gut regarding energy status. Second, it had previously been established that small molecule or peptide growth hormone secretagogues (GHSs) which activate the GHS-R also stimulated feeding [3]. Lastly, the observation that the GHS-R was strongly expressed in the NPY neurons of arcuate nucleus of the hypothalamus (ARC) [4, 5] suggested this as a potential central nervous system site of action for this peptide. In 2000, Tschop et al. [2] clearly demonstrated that ghrelin stimulated feeding and adiposity in mice and rats. Later, Cowley et al. [6] showed that ghrelin mediated activation of the GHS-R in ARC and effects which in turn modulated the electrical activity of the NPY and POMC neurons that had already been established as critical regulators of feeding behavior. These and similar results stimulated a great deal of interest because understanding the ghrelin signaling pathway was identified as a potential route in the development of successful strategies for the prevention and treatment of obesity. In spite of the overwhelming evidence that ghrelin stimulates feeding and adiposity, there is still controversy as to ghrelin's ultimate site of orexigenic activity within the CNS. This review aims to highlight some of the contentious points and to suggest some alternative explanations for the available data to this point in time.

## 2. Hypothalamic Actions of Ghrelin in the Regulation of Food Intake

The GHS-R is widely expressed in the body, being found in the brain, stomach, intestine, pancreas, heart, and gonads [7]. The density of the receptor is especially high in the pituitary, ARC, and dorsal vagal complex (DVC), including the area postrema (AP), the nucleus of the solitary tract (NTS), and the dorsal motor nucleus of the vagus (DMV) [8–12]. The high levels of expression of the GHS-R within the ARC and the fact that ghrelin-producing neurons have been discovered in the hypothalamus [6] strongly suggest that ghrelin plays a role in feeding. Indeed, a role for ghrelin in regulating food intake and energy stores draws strong support from a wide variety of different studies. Microinjections of ghrelin into the ARC as well as intracerebroventricular (ICV) injections of ghrelin strongly stimulated weight gain and feeding in rats and mice [2, 13–17]. Similar injections activated NPY and AGRP neurons within the ARC, as determined by c-Fos immunolabeling [16–18]. Ghrelin receptors are highly localized on NPY neurons [4, 5, 19], and direct application of the hormone in patch-clamp experiments increased their electrical activity [6, 20, 21]. Similar results were observed using real time quantitative Ca^++^ imaging [22] and single unit recordings [23]. Perhaps some of the strongest evidence that ghrelin regulates food intake via its action in the hypothalamus is that lesioning the ARC resulted in loss of appetite-stimulating effects of both peripheral and ICV ghrelin [24, 25]. Moreover, genetic manipulations in mice clearly show that deleting NPY reduces the effect of administered ghrelin, whereas deleting both NPY and AGRP completely abolishes the orexigenic response to ghrelin [26]. 

But does circulating ghrelin regulate energy balance as a direct consequence of primary effects on the electrical activity of ARC neurons? Although the prevailing view in the literature is a very definitive “yes”, we believe that this supposition may deserve some careful examination in view of some recent studies we will describe below. The fact that peripherally injected ghrelin not only causes weight gain and feeding [2, 15, 27, 28] but also increases c-Fos immunolabeling in the ARC, paraventricular nucleus and other areas [29–32] represent the primary data resulting in the conclusion that ghrelin directly modulates the electrical activity of GHS-R expressing neurons within the ARC by somehow crossing the blood-brain barrier (BBB), which despite alternative suggestions does exist in this region of the brain [33]. The critical issue to a true understanding of the mechanisms underlying central actions of ghrelin, we would suggest, is a clear description of how circulating ghrelin gets to and thus influences the activity of ARC and other CNS neurons involved in the regulation of energy balance. A number of different mechanisms have been suggested.

## 3. Transendothelial Cell Signaling

The neurons of the CNS are privileged in that they are protected by the BBB. It is well recognized that lipophobic molecules such as peptide hormones cannot cross the BBB by simple diffusion. Instead, such molecules can only communicate with neurons on the other side through limited routes. One route is indirect, involving binding of a circulating peptide signal to the luminal side of endothelial cells, which then produce diffusible messengers on the abluminal side [34, 35]. Gaskin et al. [36] have indeed shown that ICV injections of ghrelin stimulate synthesis of the diffusible messenger nitric oxide (NO), which in turn stimulates feeding. However in delivery by ICV injection, many experiments bypassed the function of the BBB. Thus the questions of whether and how ghrelin crosses the BBB still remain.

## 4. Specialized Transporters

Another route of communication across the BBB which has been suggested to play a role in blood-brain communication for other peptides involves the existence of specialized saturable transport mechanisms. Such transporters are able to move water soluble molecule across the BBB such that when released into the CNS side of the barrier these molecules directly contact with the neurons expressing the cognate receptors. Such transport mechanisms exist for glucose [37] and leptin although the transporter protein for leptin remains elusive (for review see [38]). Transport of circulating ghrelin across the BBB has been investigated [39–41]. Banks et al. [39] demonstrated in mice that a saturable transport system exists for transporting ghrelin from the brain to the circulation; however, no such system was identified for blood to brain transport. Interestingly, human ghrelin, which differs from mouse ghrelin by two amino acids, was a substrate for both directions of transport. Therefore while the data are suggestive, the fact that only ghrelin from another species could be transported across mouse BBB suggests that some caution should be accorded to the conclusion that a specific ghrelin transporter is responsible for transport of this peptide from the circulation to the direct milieu surrounding ARC neurons.

A further consideration with regard to the role of such a specific transporter, if it does exist, relates to the rate of transport. The plasma/CSF ratio is usually quite low, in the 4%–7% range for leptin, for example [38], and with typical concentration for ghrelin in the serum of approximately 1–3 nM [2, 15], which would translate into a transporter-mediated CSF level of about 50–150 pM. While the IC_50_ for the cloned GHS-R is reported to be 0.19–32 nM [42, 43] the observed IC_50_ values for studies examining the electrophysiological effects of ghrelin applied to neurons have been in the 1 nM to 14 nM range [44–46]. This suggests that the concentration of ghrelin found in the CSF due to a putative transport mechanism might not actually be high enough to elicit significant changes in neuronal electrical activity. Indeed, a recent investigation of plasma/CSF ratios in sheep indicated that a ten-fold increase in circulating ghrelin levels (achieved by peripheral injection) required some 40–50 minutes to increase CSF ghrelin concentrations two-fold. Moreover, the normal concentration of ghrelin in the CSF was 1000-fold lower than in the circulation [47]. Again, the CSF concentration appears for the most part to be much lower than the EC_50_ for the GHS-R.

## 5. The Blood-Brain Barrier in the Arcuate Nucleus

In the absence of a specialized mechanism to transport ghrelin, some have suggested that circulating ghrelin directly accesses ARC neurons as the result of this region being endowed with a weak or modified BBB [30, 48, 49]. Others assert that the ARC has no blood-brain barrier at all [14] or that blood borne molecules can leak from the median eminence to the ARC [32]. However, anatomical evidence indicates that the ARC demonstrates BBB integrity consistent with that found in other secure locations [33, 50–52]. In fact a classic study using IV injection of horseradish peroxidase to delineate areas with no BBB clearly indicated that the BBB of ARC is similar to that of other adjacent areas [53]. Only after eight hours of incubation is HRP staining product observable this being due to retrograde transport from axonal projections to median eminence. Further work by Broadwell et al. [54] demonstrated that IV injection of horseradish peroxidase resulted in reaction product almost entirely limited to median eminence with little evidence of entry to ARC via this route. Lastly, leakage of ghrelin from the median eminence through the parenchyma and into the ARC too is highly unlikely. The median eminence is segregated from the neuropil by a layer of tanacytes which are connected by tight junctions [55] which provide a formidable barrier against movement of solute. Also, diffusion through brain tissue is limited [38, 56], as the rate is inversely proportional to the square of the distance. 

Elevated levels of c-Fos immunostaining in the ARC after peripheral injection have been suggested to support the notion that ghrelin can cross the BBB (of the ARC) and modulate activity of neurons in ARC, which may in turn modulate activity in other brain areas [31, 32]. However, unless specifically designed to do so, neither c-Fos immunostaining studies can resolve order of activation of the areas showing increased neuronal activity nor can such studies show when ghrelin inhibits neuronal activity (see [44, 57]). Indeed, Date et al. [58] provide evidence suggesting that activation of ARC neurons and the observed increased c-Fos immunostaining in ARC induced by peripheral injection may be *secondary* to activation of vagal afferents and/or neurons within the NTS. 

While there is no doubt that neurons of the ARC and elsewhere do respond to peripheral ghrelin administration, a mechanism by which endogenous circulating ghrelin actually binds to and activates the GHS-Rs found on neurons in the ARC and elsewhere in the hypothalamus is presently unclear.

## 6. Are There Other Areas Where Ghrelin Can Directly Bind to Neurons Expressing GHS-R?

Sensory circumventricular organs (CVOs) are specialized areas of the CNS that lack the normal BBB and allow ghrelin and other peptides to bind peptidergic receptors on neurons. Unlike the capillaries of most areas of the CNS, the capillaries of the sensory CVOs have fenestrations similar to those found in the periphery, and the capillaries are not enclosed by a layer of glia cell end feet. The circulation within the sensory CVOs is also specialized to facilitate detection of circulating solutes: the capillary beds are dense and exhibit tortuosities that slow blood flow and “Virchow-Robin spaces” that allow interstitial fluid to pool around the capillaries [59] (for a complete review see [60]). Sensory CVOs are particularly well endowed with a high density and wide variety of peptidergic receptors. Lastly, the sensory CVOs have strong connections to a variety of autonomic control centers. Thus the sensory CVOs act as transducers to detect signals within the circulation and communicate this information to other centers of the CNS for integration and processing. There are three sensory CVOs: the subfornical organ (SFO), area postrema (AP), and the organum vasculosum of the lamina terminalis (OVLT). Both the SFO and the AP are known to express the GHS-R and to contain ghrelin-sensitive neurons; there have not been data suggesting that the OVLT plays a role in the detection of ghrelin. 

The SFO projects from the rostral wall into the third ventricle and is located dorsally to the lamina terminalis. It sends direct and indirect projections to vasopressin and oxytocin neurons of the paraventricular nucleus and the supraoptic nucleus of the hypothalamus [61]. The SFO also sends projections to the parvocellular neurons of the PVN [62], the ARC [63, 64], and other areas of the hypothalamus. Afferent projections to the SFO include the NTS, the lateral hypothalamus, the midbrain raphe, and other areas [64–66]. Thus, the SFO is in direct contact with ghrelin in the circulation and communicates with key autonomic control centers.

The AP is located in the fourth ventricle, on the dorsal surface of the medulla positioned adjacent to the NTS. The AP together with the NTS and DMV makes up the dorsal vagal complex, a major site for integration of afferent information from the gut, viscera, and circulation. The AP has reciprocal connections with a variety of targets; most notably are the NTS, the lateral parabrachial nucleus, the nucleus ambiguus, and the tegmental nuclei [67, 68]. There are also descending connections from the PVN to AP [69]. Of particular interest, however, both the NTS and lateral parabrachial nucleus send ascending connections to the PVN and ARC [70–75]. Like the SFO, the neurons of the AP are in direct contact with ghrelin and other hormones in the circulation and communicate with autonomic control centers. 

There is already an established body of evidence describing the roles of two of the sensory CVOs: the subfornical organ (SFO) and the area postrema (AP) in homeostatic regulation. For example, the SFO is well known to regulate fluid balance via angiotensin II signaling, and AP is well known to play critical roles in cessation of feeding via the anorectic hormones: CCK and amylin (for reviews see [60, 76–78]). The remainder of this review will focus on the roles of SFO and AP in the processing of peripheral ghrelin signals.

## 7. Subfornical Organ Actions of Ghrelin

**Figure 1:**
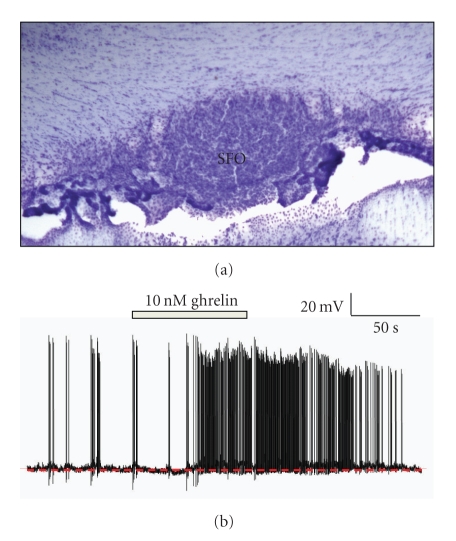
Ghrelin modulates the electrical activity of SFO neurons. (a) Nissl-stained coronal section showing the SFO. (b) Representative current-clamp recordings from dissociated SFO neurons that were depolarized by application of 10 nM ghrelin. This effect is mediated by activation of a cation conductance. Interestingly, separate populations of SFO neurons depolarize in response to either ghrelin or amylin (not shown). No SFO neurons are sensitive to both ghrelin and amylin; see [45].

Some of the first evidence that ghrelin may play a role in regulation of energy balance by action at the SFO came in 2006 when Pulman et al. [45] demonstrated that the GHS-R was expressed in SFO and that a subpopulation of SFO neurons was dose dependently excited by the application of exogenous ghrelin. The same study revealed that amylin, an anorexigenic peptide, also excited a subpopulation of SFO neurons and intriguingly the subpopulations of ghrelin-sensitive and amylin-sensitive neurons were mutually exclusive (Figure 1). This suggested that orexigens and anorexigens both acted at the SFO, but via different neuronal pathways. These data are supported by the observation of Takayama et al. [32] who observed increased c-Fos staining in the SFO after peripheral ghrelin administration. 

The SFO is classically thought of as a key CNS site controlling thirst and drinking (for review see [77]), and studies now also suggest that this CVO is a key site of action for regulation of not only food but also water intake by ghrelin. Two groups gave ICV injections of ghrelin into rats and observed that while ghrelin increased food intake, it also inhibited water intake [79, 80], effects which were also observed following peripherally injected ghrelin [81]. While ghrelin is primarily known for its actions on food intake, these recent observations underscore the notion that food and water intakes are both ingestive behaviors and are inextricably linked (for review see [82]).

## 8. Area Postrema Actions of Ghrelin

While a potential role for ghrelin in regulating energy balance at the AP has long been recognized (for review see [82, 83]), it has not received the same attention as the signaling pathways including NPY/AGRP and POMC neurons within the ARC (for a review of the notion of the ARC-centric versus distributed network hypotheses see [84]). The GHS-R is expressed in the AP [9], strongly suggesting a role for ghrelin in the regulation of AP neuronal function, a conclusion supported by studies showing that peripheral administration of ghrelin caused increases in c-Fos immunoreactivity in AP, DMV, and NTS [32, 81, 85]. This result was not surprising given the unfettered access of circulating ghrelin to AP neurons. Importantly, ablation of AP resulted in the loss of c-Fos immunostaining in DMV and NTS, suggesting that ghrelin acts directly at the AP, while acting indirectly at the DMN and NTS [85]. The effect on c-Fos staining in ARC after AP lesion and peripheral injection of ghrelin is currently unknown. 

Peripheral injection of ghrelin can stimulate feeding almost instantly [47]; therefore, the speed of the detection of ghrelin signals is a point of interest. One may hypothesize that the brainstem connection to ARC, in spite of being polysynaptic, is probably faster and more consistent with latency to feeding than to the inefficient putative transport of ghrelin across the BBB [47]. Unfortunately, there are not sufficient c-Fos immunostaining data to compare the relative time course for activation of AP and NTS to the activation of ARC. 

Recent observations by Gilg and Lutz [86] provide additional strong support for a central role for the AP in ghrelin-mediated feeding. They observed that lesion of AP eliminated ghrelin-induced feeding in two separate administration paradigms although an increase in body weight was still observed, likely because of the ability of ghrelin to reduce fat utilization [2]. This observation conclusively demonstrates that ghrelin-induced feeding critically depends on intact signaling at the AP. In further support of the notion that the caudal brainstem plays a pivotal role in ghrelin-induced feeding, Date et al. [58] carried out experiments lesioning the midbrain of rats to destroy NTS-ARC projections. They observed that the lesion abolishing communication between the NTS and the hypothalamus eliminated both feeding and c-Fos immunostaining in the ARC that are normally induced after peripheral injection of ghrelin. Importantly, in these studies feeding in response to ICV-administered ghrelin remained intact after the midbrain lesion, showing that hypothalamic and brainstem responses to ghrelin are separable, yet linked. Date et al. [58] argue in favor of a model where the vagus nerve detects ghrelin and transmits this information to the ARC via noradrenergic projections from the NTS. The role of vagal afferent signaling has been questioned by Arnold et al. [87] and the noradrenergic nature of the NTS efferents by Faulconbridge et al. [88]. Still, the Date model is not inconsistent with the possibility that information regarding endogenous circulating ghrelin gathered by AP and vagus is integrated within the NTS then communicated to the ARC for further integration. Clearly, the role of the AP and caudal brainstem cannot be overlooked. 

Another work provides strong support for the role of the caudal brainstem in ghrelin-mediated feeding. Faulconbridge et al. observed that direct injection of ghrelin into the caudal brainstem elicits feeding at similar or lower concentrations than required for ARC microinjections [89] and that the food-stimulating doses in caudal brainstem do not activate c-Fos in ARC [88]. The same group proposes a strong contribution of caudal brainstem neurons expressing NPY1 receptors [90]. While their data are not in complete agreement with Date et al. [58] regarding the role of an aminergic NTS connection to ARC, they do strongly support the notion that caudal brainstem and ARC are ghrelin-responsive areas with each contributing to ghrelin-mediated feeding.

**Figure 2:**
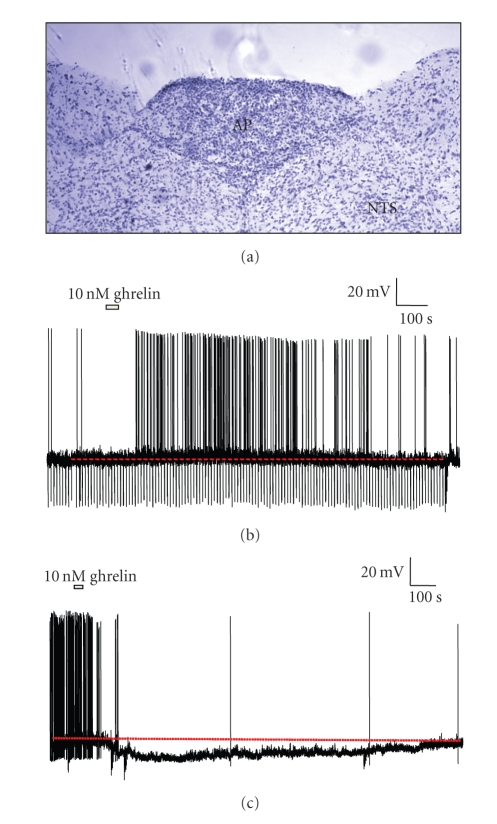
Ghrelin modulates the electrical activity of AP neurons. (a) Nissl-stained coronal section showing the AP, along with surrounding areas including NTS. ((b) and (c)) Representative current-clamp recordings from dissociated AP neurons that were depolarized or hyperpolarized by focally applied 10 nM ghrelin. The electrophysiological effects were caused by activation of a cation conductance (depolarization) or activation of K^+^ current (hyperpolarization); see [44].

Towards understanding the role of AP in ghrelin signaling, Fry and Ferguson [44] examined the sensitivity of AP neurons to ghrelin using patch-clamp analysis. These experiments demonstrated that about 40% of neurons within the AP were sensitive to ghrelin, exhibiting changes in electrical properties, and firing rates when exposed to the hormone. Intriguingly, half of these showed increased excitation, and the other half showed reduced excitation in response to application of physiologically relevant concentrations of ghrelin (Figure 2). The observation that cells can be inhibited by ghrelin may suggest why some studies have not observed an increase in c-Fos staining in the AP after administration of ghrelin [31]. Further experiments to investigate the projections and network properties of the ghrelin-sensitive AP neurons are ongoing.

## 9. Conclusions

To reiterate, there is no doubt that ARC plays a role in the regulation of energy balance by the hormone ghrelin. However, there are sufficient reasons to think that the activity of ghrelin on energy balance may not be localized entirely to the ARC but is distributed across different brain regions. In particular, the sensory CVOs are well positioned detecting circulating ghrelin and passing this information to other integrative centers for additional processing. Based on recent observations, the sensory CVOs appear to play a crucial role in the signaling pathways of ghrelin. Understanding the interaction between the sensory CVOs, the ARC, and many other CNS nuclei implicitly linked to the regulation of energy balance will hopefully lead us towards developing successful new strategies for the prevention and treatment of obesity.
